# Identification of Novel Immunogenic Proteins of *Neisseria gonorrhoeae* by Phage Display

**DOI:** 10.1371/journal.pone.0148986

**Published:** 2016-02-09

**Authors:** Daniel O. Connor, Jonas Zantow, Michael Hust, Frank F. Bier, Markus von Nickisch-Rosenegk

**Affiliations:** 1 Department of Bioanalytics and Biosensorics, Fraunhofer Institute for Cell Therapy and Immunology, Branch Bioanalytics and Bioprocesses (IZI-BB), Potsdam, Germany; 2 Institute for Biochemistry, Biotechnology and Bioinformatics, Technische Universität Braunschweig, Braunschweig, Germany; 3 Institute of Biochemistry and Biology, University of Potsdam, Potsdam, Germany; 4 Department of Biosystem Integration and Automation, Fraunhofer Institute for Cell Therapy and Immunology, Branch Bioanalytics and Bioprocesses (IZI-BB), Potsdam, Germany; Centro Nacional de Biotecnologia—CSIC / CIF Q2818002D, SPAIN

## Abstract

*Neisseria gonorrhoeae* is one of the most prevalent sexually transmitted diseases worldwide with more than 100 million new infections per year. A lack of intense research over the last decades and increasing resistances to the recommended antibiotics call for a better understanding of gonococcal infection, fast diagnostics and therapeutic measures against *N*. *gonorrhoeae*. Therefore, the aim of this work was to identify novel immunogenic proteins as a first step to advance those unresolved problems. For the identification of immunogenic proteins, pHORF oligopeptide phage display libraries of the entire *N*. *gonorrhoeae* genome were constructed. Several immunogenic oligopeptides were identified using polyclonal rabbit antibodies against *N*. *gonorrhoeae*. Corresponding full-length proteins of the identified oligopeptides were expressed and their immunogenic character was verified by ELISA. The immunogenic character of six proteins was identified for the first time. Additional 13 proteins were verified as immunogenic proteins in *N*. *gonorrhoeae*.

## Introduction

*Neisseria gonorrhoeae* is a gram-negative pathogenic β-proteobacterium, which usually appears as diplococci and is strictly limited to the human as host. It belongs to the genus *Neisseria* that also includes the human pathogen *Neisseria meningitidis* and several non-pathogenic species, e. g. *Neisseria lactamica* or *Neisseria cinerea*. *N*. *gonorrhoeae* is often abbreviated as gonococcus and is the causative agent of gonorrhoea, one of the most common sexually transmitted infections (STI) with an estimated number of 106 million new infections worldwide in 2012 [1]. It colonizes the urogenital epithelial cells and can shift to the upper genital tract which can lead to pelvic inflammatory disease (PID) in women [2,3]. In rare cases, gonococcal infection can cause sterility in men and infertility in women as implications of PID [4]. Furthermore, recent studies mention that a gonococcal infection increases HIV transmission rates [5,6]. Given that around 80% of gonococcal infections in women [4] and to a lesser extent in men [7–10] are asymptomatic, treatment as well as diagnosis are hindered.

Since the advent of antibiotic treatment of *N*. *gonorrhoeae*, it has been a challenge to stay ahead of gonococcal evolution. Due to the pathogen’s exceeding ability for DNA and gene uptake, it has been able to develop resistances to various formerly used antibiotic classes, e. g. sulfanilamides, penicillins, tetracyclines, and most recently fluoroquinolones [6,11,12], potentially leading to an evolution of a future “superbug”. Lately, resistant strains against the last line of defence—the cephalosporins such as ceftriaxone—were isolated in different parts of the world [13–15]. Hence, a better understanding of *N*. *gonorrhoeae* infection and virulence could facilitate new treatments of gonorrhoea. Elucidation of novel immunogenic proteins could provide first indications of crucial proteins involved in gonococcal pathogenicity.

Another application for immunogenic proteins is the development of vaccines. Due to a multiplicity of evasion mechanisms to circumvent the immune response, e. g. antigenic variability of surface structure [16,17] as well as production of IgA_1_ proteases [18,19], there is only a minimal antibody generation detectable after infection. Furthermore, the immune response primarily occurs at the mucosal level that is not sustained leading to potentially high reinfection rates even with the same strain [15]. For these reasons, the development of a vaccine is complex and is not in immediate sight [10,12]. Nevertheless, by examination and determination of novel immunogenic proteins specific ways for treatment of *N*. *gonorrhoeae* could be possibly found, for instance, by inactivating virulence associated proteins preventing infection or by specifically disturbing essential cell processes.

On top of that, immunogenic proteins could be used for developing diagnostic tools as an easy-to-use system. State of the art in gonococcal diagnosis is the isolation and cultivation followed by gram stain to identify *N*. *gonorrhoeae* and to simultaneously screen for antibiotic resistances [4]. Drawbacks are long incubation times and insensitivity to asymptomatic infections [20]. Possible alternatives include nucleic acid amplification tests (NAATs), which have many advantages, e. g. easy specimen collection, insusceptible transportation, and time savings, but still have to be conducted by laboratory personnel and cannot distinguish between different antibiotic resistances [15,21]. Since a large number of infections occur asymptomatic and many people suffering from STIs feel ashamed to see a physician, it would be a great benefit to have an easy-to-use and inexpensive diagnostic tool such as a urine test strip to identify *N*. *gonorrhoeae* cells in urine. Due to antigenic variability and a limited knowledge, there is a lack of suitable proteins. Hence, these challenges might be overcome by identifying novel immunogenic proteins as targets for the development of detection methods or for vaccine research. Furthermore, the identified proteins might elucidate mechanisms of gonococcal infection. Commonly, the identification of immunogenic proteins is performed by 2D-PAGE and immunoblot followed by mass spectrometry [22–25], by time and labour consuming expression of the proteome and screening on nitrocellulose membranes [26], or recently by microarray technique [27–29]. However, all of these methods have certain disadvantages. Approaches with 2D-PAGE and expression libraries with cDNA have limitations in detecting differentially expressed proteins, e. g. in host-pathogen interaction, weakly expressed proteins or proteins with a molecular weight below 10 kDa [30–32]. Expression libraries of the complete proteome entail complex production and purification procedures. Furthermore, screenings of thousands of clones have to be accomplished. In contrast, phage display of antigens may avoid these limitations. Phage display technology was invented by George P. Smith thirty years ago [33]. This technology is mainly used for antibody selection [34–36] but has also been applied to identify immunogenic proteins from genomic and cDNA libraries [37].

In the pHORF approach, genomic libraries of pathogens are cloned in phagemids and packaged with Hyperphage [38,39] for ORF selection to improve the library quality [40]. Oligopeptides or protein domains are displayed on the surface of the M13 phage particles and the corresponding genetic information on the phagemid is packaged in the phage particles. Thus, the phenotype (protein fragments) is coupled with the genotype (corresponding gene fragments). By using polyclonal antibodies, patient or animal sera, immunogenic oligopeptides are selected in a panning procedure [41]. This technology allows for the discovery of immunogenic proteins independent from pathogen cultivation, thus also identifying proteins only expressed in the patient (in vivo host pathogen context) [37]. This method has been used successfully to identify a list of novel biomarkers from *Salmonella* Typhimurium and *Mycoplasma hyopneumoniae* [41–43]. The technology was also used to identify biomarkers of ticks [44]. Compared to competing technologies like 2D-PAGE of proteins from cultivated pathogens followed by immunoblot using sera and mass spectrometry, the pHORF technology also allows identifying proteins expressed only in host-pathogen interaction and proteins smaller than 10 kDa. Other methods such as SEREX [45] working with lytic T7 phage or phage display systems also using M13 do not have a simple open reading frame enrichment to increase the library quality.

In this study, we successfully constructed a phage display library from the whole genome of *N*. *gonorrhoeae* with a coverage of more than 100 fold compared to the calculated theoretical number of inserts. Through two to three consecutive rounds of panning and final screening with polyclonal rabbit antibodies to *N*. *gonorrhoeae*, 21 potentially immunogenic oligopeptides were identified. Thirteen corresponding proteins were expressed in full-length and the immunogenic character of 11 thereof was verified. Six full-length proteins were identified as immunogenic for the first time; most of the remaining identified oligopeptides have been described in literature within the closely related pathogen *Neisseria meningitidis*. The immunogenic character of these proteins was validated for *N*. *gonorrhoeae* in this study. Furthermore, the proteins were modelled and the phage displayed oligopeptides were mapped to the obtained models, indicating those parts of the protein with higher immunogenic character. The obtained immunogenic proteins could serve as promising candidates for the further clarification of gonococcal infection and for the development of effective agents against *N*. *gonorrhoeae*.

## Results

### Construction of a *N*. *gonorrhoeae* genomic phage display library

**Fig 1:**
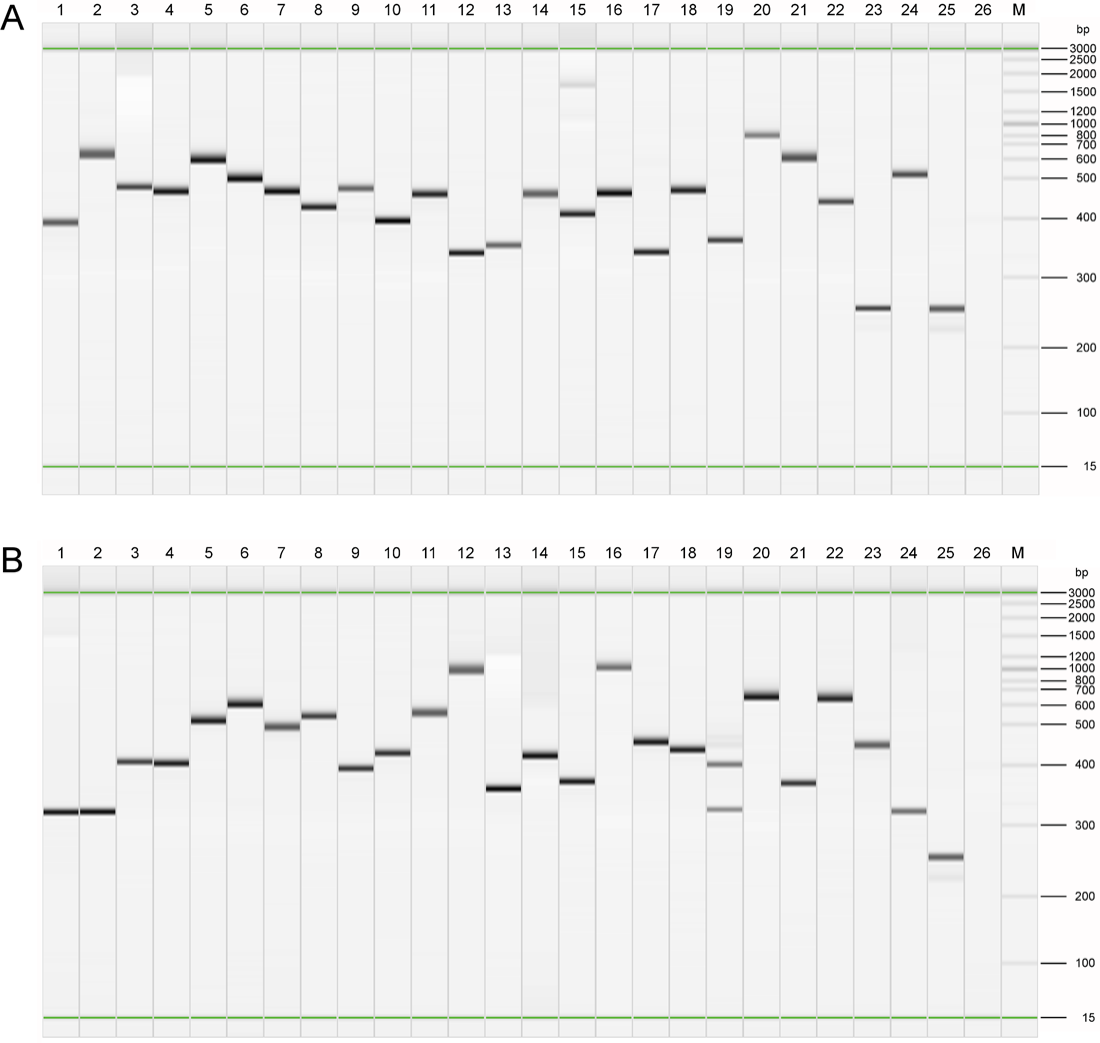
Colony PCR results presented as gel images. **A** cloned library fragments **B** Hyperphage packaged inserts. Lanes 1–24 randomly picked clones from three different libraries (A) and from the phage packaged library (B), Lane 25 Empty pHORF3 vector control without insert, Lane 26 No template control, M Size ladder.

Genomic DNA of *N*. *gonorrhoeae* was fragmented by sonication and cloned into pHORF3, resulting in three libraries with 1.3 x, 1.0 x and 1.2 x 10^7^ independent clones with an average insert size of 190 bp, 290 bp and 174 bp, respectively. The libraries covered the theoretically required number of clones of 7.8 x, 5.1 x and 8.55 x 10^4^ more than 100 fold. The insert rates and sizes were analysed by colony PCR (Fig 1A) and sequencing. Eight randomly picked clones of each library were used for colony PCR and an additional seven per library were picked for sequencing. All libraries had insert rates of more than 85%. The inserts ranged from 56 bp to 450 bp. The libraries were then pooled resulting in an average insert length of 218 bp and used for Hyperphage packaging. The packaged library was checked for the number of inserts by colony PCR (Fig 1B) and for correct in-frame inserts by sequencing. While the cloned libraries had an in-frame insert ratio of approximately 5%, the in-frame insert rate was increased to 70% after Hyperphage packaging. The average insert size was 216 bp.

### Panning and screening of oligopeptide phage

A polyclonal rabbit antibody to *N*. *gonorrhoeae* was used for the panning rounds. Individual clones were picked after two and three panning rounds. Clones were picked after two and three rounds of panning since binders with low affinities are removed during the panning process resulting in only the best binders after three consecutive panning rounds. This is advantageous for isolation of recombinant antibodies; however, in case of immunogenic proteins it would be ideal to identify all proteins with immunogenic character regardless of their affinity. In total, 276 oligopeptide phage clones were produced and analysed by ELISA. The average value of the negative controls was subtracted from all values and clones with higher signals than 0.3 were further analysed by sequencing.

Twenty-one different genes were identified and checked for similarity by BLAST (Table 1). Furthermore, phage particles presenting different parts of the same gene were identified (exemplary for NGO0592 see S1 Fig.). Thirteen of the identified proteins have been described in the literature before. Eight oligopeptides exhibited no homologies to known antigens. One clone carried parts of two genes—NGO1656 and NGO1657—the last 92 bp of NGO1657, a non-coding part of 48 bp and 131 bp of NGO1656. Both genes were chosen to further characterise the immunogenic character of their encoded proteins. The eight potential immunogenic proteins and several controls were chosen for full-length protein production (Table 1). Additionally, NGO1500 (glutamate racemase) was obtained through panning; however, it was dismissed after screening due to low signal intensities in ELISA and used as a negative control.

**Table 1:** Obtained oligopeptides isolated by phage display and corresponding proteins identified by BLAST.

*N*. *gonorrhoeae* locus tag	Protein name	Number of inserts found	Insert size (bp)	Protein mass of the complete protein (kDa)	Homologue in *N*. *meningitidis*	Localisation prediction by PSORTb	Previously identified as immunogenic in *Neisseria* genus or found in outer membrane vesicle vaccines	Reference
NGO0170*	ABC transporter, ATP-binding protein	1	121	28.12	NMB0588	Cytoplasmic membrane	No	-
NGO0326*	RNA-binding protein Hfq	1	109	10.78	NMB0748	Cytoplasm	No	-
NGO0451*	replicative DNA helicase	1	76	52.18	NMB0885	Cytoplasm	No	-
NGO0564*	dihydrolipoamide acetyltransferase	1	310	54.69	NMB1342	Cytoplasm	Yes	[46]
NGO0584	50S ribosomal protein L9	2	130/148	15.07	NMB1320	Cytoplasm	Yes	[47]
NGO0592	trigger factor	3	115–208	48.37	NMB1313	Cytoplasm	Yes	[48]
NGO0642*	tRNA pseudouridine synthase B	1	262	33.39	NMB1374	Cytoplasm	No	-
NGO0777*	DNA-binding protein Hu	1	100	9.38	NMB1230	Cytoplasm	No	-
NGO0916*	dihydrolipoamide succinyltransferase	1	272	41.69	NMB0956	Cytoplasm	Yes	[49]
NGO0983	outer membrane protein H.8	1	174	8.02	NMB1523	Outer membrane	Yes	[50]
NGO1043*	hypothetical protein	2	160/217	11.41	NMB1468	Periplasm	Yes	[51]
NGO1429	molecular chaperon DnaK	10	67–325	68.86	NMB0554	Cytoplasm	Yes	[25,48]
NGO1577	outer membrane protein PIII	6	115–274	25.54	NMB0382	Outer membrane	Yes	[48,52]
NGO1634*	hypothetical protein, putative phage associated protein	2	97/133	21.11	-	Cytoplasm	No	-
NGO1656*	conserved hypothetical protein	1	271	31.53	NMB0345	Unknown, non-cytoplasmic	Yes	[48,53]
NGO1657*	stress-induced morphogen BolA		insert NGO1556	10.02	NMB0344	Unknown, non-cytoplasmic	No	-
NGO1796*	ribosome recycling factor	1	295	20.64	NMB0187	Cytoplasm	No	-
NGO1852*	50S ribosomal protein L7/L12	3	136–289	12.56	NMB0131	Cytoplasmic membrane/ Periplasm	Yes	[25]
NGO2094	chaperonin 10 kDa subunit	5	103–196	10.29	NMB1973	Cytoplasm	No, but identified in other bacteria	[54,55]
NGO2095	chaperonin 60 kDa subunit	11	67–301	57.35	NMB1972	Cytoplasm	Yes	[25,48]
NGO2139	genome-derived Neisserial antigen 1946	1	327	31.34	NMB1946	Cytoplasmic membrane	Yes	[56,57]

The number of inserts found during screenings with different insert sizes and presenting different parts of the protein are listed. Proteins with locus tags marked with an asterisk (*) were chosen for full-length production to verify their immunogenic character.

### Cloning and production of full-length proteins

**Fig 2:**
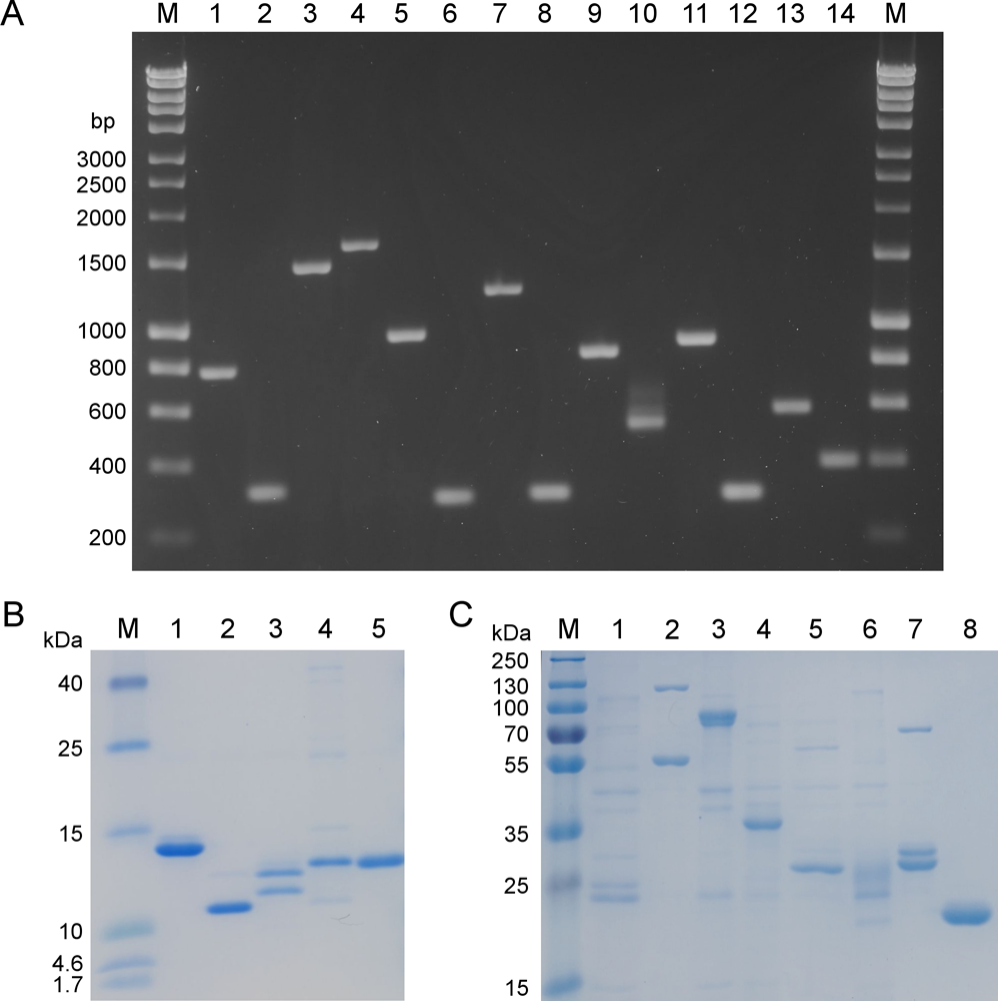
Cloning and protein expression of full-length genes and full-length proteins. **A** 1.5% agarose gel: M Hyperladder I (bioline, Bio-33025-26), 1 NGO0170, 2 NGO0326, 3 NGO0451, 4 NGO0564 (+ control), 5 NGO0642, 6 NGO0777, 7 NGO0916, 8 NGO1043, 9 NGO1500 (- control), 10 NGO1634, 11 NGO1656, 12 NGO1657, 13 NGO1796, 14 NGO1852. **B** SDS-PAGE (15%) of 0.5 μg each of produced proteins: M Spectra Multicolor Low Range Protein Ladder (Thermo Scientific, 26628), 1 NGO0326, 2 NGO0777, 3 NGO1043, 4 NGO 1657, 5 NGO1852. **C** SDS-PAGE (10%) of 0.5 μg each of produced proteins: M PageRuler Plus Prestained Protein Ladder (Thermo Scientific, 26619), 1 NGO0170, 2 NGO0451, 3 NGO0564 (+ control), 4 NGO642, 5 NGO1500 (- control), 6 NGO1634, 7 NGO1656, 8 NGO1796.

In total, 15 proteins were chosen for recombinant expression in *Escherichia coli* (the 13 proteins labelled with an asterisk in Table 1, NGO1500 and BB0069 of *Borrelia burgdorferi* as negative controls). All identified oligopeptides which were not described as immunogenic in literature before were produced as full-length proteins (compare Table 1). Furthermore, three proteins known to have an immunogenic character in *N*. *meningitidis* with localisations predicted as non-cytoplasmic were expressed in full-length. However, proteins that were known to be immunogenic in many other bacteria or proteins which had been investigated to a high extent in *N*. *meningitidis* were not produced as full-length proteins. All full-length genes were amplified (Fig 2A) and positive clones were identified by colony PCR and sequencing after transformation. All proteins except NGO0916 (selected as a positive control that was isolated from outer membrane vesicle vaccines of *N*. *lactamica* and *N*. *meningitidis* [49]) were expressed and purified by affinity chromatography using the polyhistidine-tag of the proteins (Fig 2B and 2C). NGO0564 was applied as a positive control, since it was also found in outer membrane vesicle vaccines of *N*. *lactamica* and *N*. *meningitidis* [49]. NGO1043 was expressed harbouring a pelB leader sequence with a length of 21 amino acids. Proteins with an apparent molecular mass of 11 kDa and 13 kDa were detected by SDS-PAGE; the latter corresponding to NGO1043 plus leader sequence, the smaller band corresponding to NGO1043 alone. The production of recombinant NGO1656 resulted in two different variants that arose from a predicted signal sequence of 20 amino acids.

### Validation of immunogenicity of the full-length proteins

**Fig 3:**
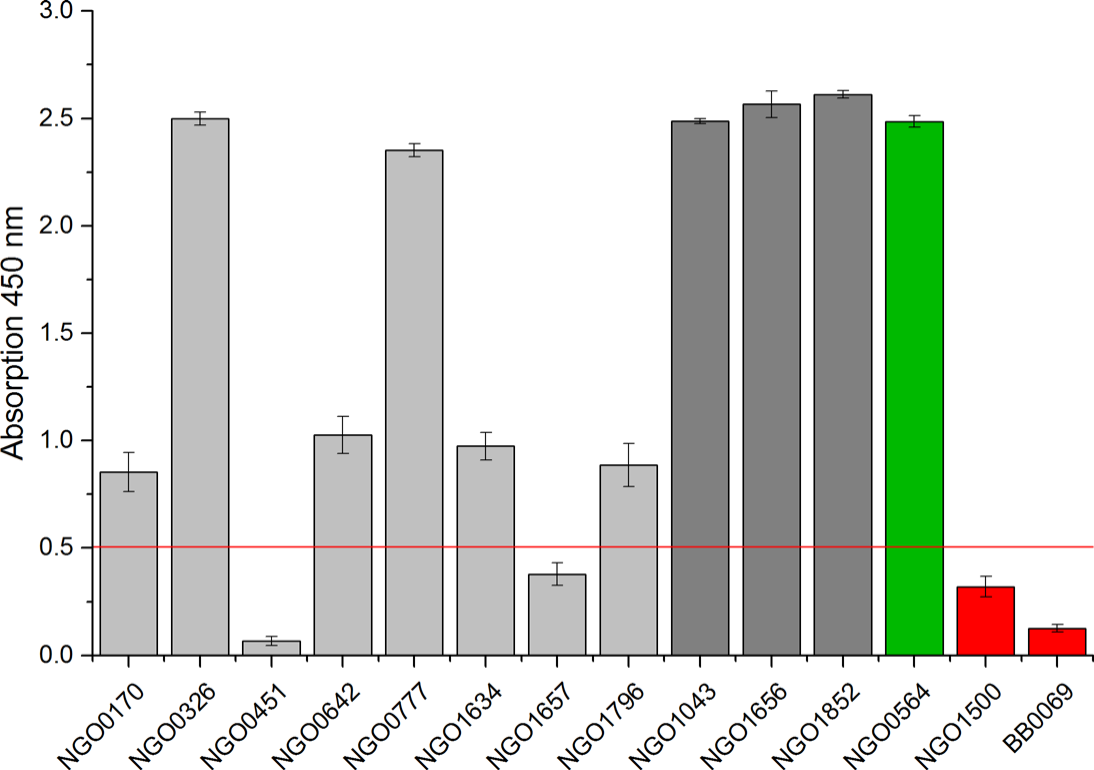
ELISA of expressed and purified full-length proteins to verify their immunogenic character (n = 3). Three polyclonal rabbit antibodies against *N*. *gonorrhoeae* from different immunisations were used. Two proteins were applied as negative controls (red columns): Protein BB0069 from *B*. *burgdorferi* that was expressed and purified under identical conditions and NGO1500, a glutamate racemase. The cut-off was set to fourfold of the signal of the negative control BB0069. Blank intensity of a buffer control was subtracted from each sample. NGO0564 was applied as positive control (green column). Investigated proteins of which the immunogenic character had previously been unknown are coloured in light grey. Proteins that are known to have an immunogenic character in *N*. *meningitidis* are coloured in dark grey.

The expressed full-length antigens were validated by ELISA using three commercially available polyclonal antibodies from different immunisations (Fig 3). NGO0564, NGO1043, NGO1656 and NGO1852 were detected with high signals in ELISA. Proteins NGO0170, NGO0326, NGO0642, NGO0777, NGO1634 and NGO1796 had ELISA signals above the cut-off. However, NGO1657 was below the cut-off. Furthermore, NGO0451 had the lowest signal intensity of all samples in ELISA. NGO1500 and an expressed protein from *Borrelia burgdorferi* (BB0069) were applied as negative controls. NGO1500 was selected after three consecutive rounds of panning but showed low signal intensity in screening ELISA. Hence, the cut-off was set to fourfold of the signal intensity of BB0069 after the blank signal intensity of a buffer control was subtracted.

### Real-time PCR for verification of NGO1634 expression

To verify the protein expression of the putative phage associated protein (NGO1634) or rather the RNA transcription of its gene, a real-time PCR was conducted. The RNA integrity number (RIN) of each RNA sample was determined. The lowest RIN value for the three isolated RNAs was 8.9. A real-time PCR (S2 Fig) with the three cDNAs and corresponding negative controls yielded the following average crossing points: 20.94 ± 1.7 for NGO0715 (gdh, positive control), 27.25 ± 0.82 for NGO1634 (sample) and > 35 for the no template controls and all other negative controls (RT-, NTC of each sample).

### Mapping to protein models

**Fig 4:**
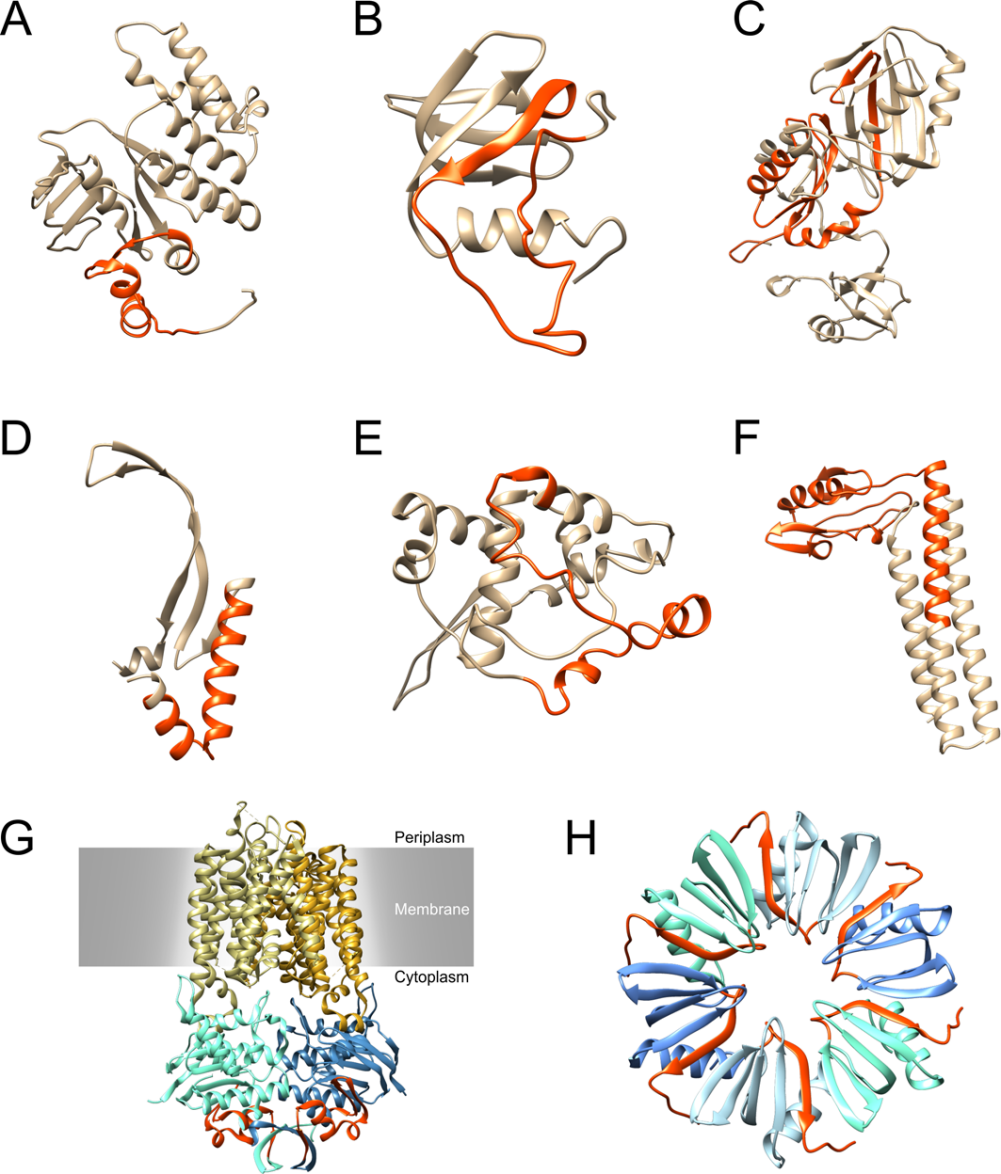
Mapping of the isolated phage displayed oligopeptides to the full-length protein models. Phage selected oligopeptides are highlighted in orange-red. Proteins that are likely to be multimeric or are located in membranes are additionally displayed as their multimeric homologues. **A-F** Models of monomeric proteins: **A** NGO0170, **B** NGO0326, **C** NGO0642, **D** NGO0777, E NGO1634, **F** NGO1796. **G** Multimeric model of the closest homologue to NGO0170 determined by I-TASSER, a HI1470/1 transporter from *H*. *influenza*, embedded in the cytoplasmic membrane [62]. **H** Multimeric model of the closest homologue to NGO0326 determined by I-TASSER, a RNA-binding Hfq protein from *L*. *monocytogenes* [63].

Protein models were predicted for all novel immunogenic proteins using I-TASSER [58–60]. PSI-BLAST [61] was applied with three iterations to search for homologues in the PDB database. A summary of the best hits for each protein can be found in the supplementary section (S1 Table). The identified oligopeptides isolated by phage display were then mapped to the protein models in monomeric shape (Fig 4). For multimeric proteins the corresponding oligopeptide was also highlighted in the closest homologue to gain insight into accessibility of the immunogenic region. For NGO0170, predicted to be located in the cytoplasmic membrane, the identified oligopeptide is located at the C-terminal end of the protein spanning over three α-helices and two β-sheets. The closest structural homologue to NGO0170 is a hetero tetramer of *Haemophilus influenza* consisting of two identical proteins spanning the membrane and two identical proteins which extend into the cytoplasm [62].

The oligopeptide identified from NGO0326 is also located at the protein’s C-terminus including one α-helix and one β-sheet. The closest structural homologue to NGO0326 was a homo hexamer of *Lysteria monocytogenes* which is most likely organised in a cyclic assembly illustrated in Fig 4H [63]. Oligopeptides from NGO0642 and NGO0777 are identified in the N-terminal part of the proteins while NGO1634 and NGO1796 exhibit oligopeptides in the middle of the protein. All models were checked for their reliability by QMEAN [64]. QMEAN scores were as follows: NGO0170 0.652, NGO0326 0.630, NGO0642 0.633, NGO0777 0.586, NGO1634 0.340, NGO1796 0.675. Low QMEAN score and I-Tasser's C-score for the NGO1634 model indicate its low reliability for implying the protein's structure and function.

## Discussion

Several immunoproteome studies have been conducted to investigate immunogenic proteins of *N*. *meningitidis* [25,48,65,66], a human pathogen closely related to *N*. *gonorrhoeae*. Furthermore, reverse vaccinology has been applied to identify antigens from *Neisseria* strains included in effective outer membrane vesicle vaccines [47,49]. In contrast, immunoproteome studies of *N*. *gonorrhoeae* have not been available yet. In this work, we conducted an immunoproteomic approach to identify novel immunogenic proteins of *N*. *gonorrhoeae*. Due to low antibody titers in humans, no human sera are accessible. Hence, polyclonal rabbit antibodies to *N*. *gonorrhoeae* were applied for panning and screenings. The screening of a pHORF phage display library successfully yielded a number of previously unknown immunogenic proteins. Identification of novel immunogenic proteins might grant additional insight into their role in pathogenicity and virulence of *N*. *gonorrhoeae*. Furthermore, proteins involved in the pathogenic pathway might serve as potential candidates for therapeutic treatments.

Altogether, 21 oligopeptides were identified by phage display screening with a polyclonal rabbit antibody to *N*. *gonorrhoeae* FA 1090. Furthermore, in NGO0592, NGO1429, NGO1577, NGO2094 or NGO2095 several different oligopeptides covering diverse parts of these proteins were identified. This indicates that numerous parts of said proteins were recognised as potential epitope regions.

Thirteen oligopeptides were produced as full-length proteins to validate their immunogenic character or to serve as a control. Six of those proteins had not been identified as immunogenic before and their immunogenic character was successfully validated by ELISA: NGO0170 is an ABC transporter ATP-binding protein that is involved in the transport of zinc/manganese. The localization is predicted to be in the cytoplasmic membrane. The phage displayed oligopeptide is located at the C-terminal end of the protein indicating the immunogenic part of the protein. The closest homologous structure identified by I-TASSER was a putative metal-chelate type ABC transporter of *Haemophilus influenza* [62] which occurs as hetero tetramer with two identical chains each. By comparing the query and aligned sequence, the oligopeptide identified by phage display was mapped to the part of the protein that is not embedded in the membrane (Fig 4G). Therefore, the identified oligopeptide is accessible by antibodies raised against it. On the other hand, it is most likely that the protein is facing inward to the cytoplasm hindering the accessibility of antibodies from the outside. However, localization and orientation are only predictions making it necessary to further investigate NGO0170. Interestingly, homologies on the amino acid level between NGO0170 (NMB0588) and an ORF of a potential pathogenicity island of *E*. *coli* have been described [67] underlining the possible influence of NGO0170 on the pathogenicity of *N*. *gonorrhoeae*. Furthermore, the homologue in *N*. *meningitidis* (NMB0588) was found to be upregulated at low zinc levels [68]. This condition often occurs during infection of the human host cells. A deletion mutant lacking NGO0170 could provide further information on the importance of this protein for the viability and virulence of *N*. *gonorrhoeae*. Depending on the impact, NGO0170 could be an attractive target for a novel therapeutic treatment for *N*. *gonorrhoeae*. NGO0326 is a highly conserved host factor-I binding protein abbreviated Hfq. Its function is the modulation of posttranscriptional regulation in stress response as a RNA chaperone. It is known to play a vital role in the virulence of different bacteria [69–71] including *N*. *meningitidis* [72]. Furthermore, it has been shown that a deletion of the homologue of NGO0326 in *N*. *meningitidis* leads to a differentiated expression of 369 ORFs [73]. This resulted in an altered bacterial growth rate and reduced adherence to epithelial cells. NGO0326 showed very high signal intensities in the range of the positive controls. Homologues of NGO0326 were shown to take a cyclic hexameric shape. The closest homologous structure identified by I-TASSER was the Hfq protein of *L*. *monocytogenes* [63]. Sequence coverage was 75%. However, the last 24 amino acids of the NGO0326 sequence were not covered by the closest structural homologue. The identified oligopeptide spanned precisely 35 of the last 39 amino acids at the C-terminal end of the protein, though. Hence, the larger part of the identified oligopeptide was not included by the predicted model and its homologous structures. Since NGO0326 is located in the cytoplasm, the oligopeptide should be accessible if the cell and membrane are disrupted. NGO0642 is annotated as a tRNA pseudouridine synthase B. It is responsible for the synthesis of pseudouridine utilized in tRNAs and is highly conserved in *Neisseria* species with 80%–96% identity. The closest structural homologue identified by I-TASSER was a tRNA pseudouridine synthase TruB from *E*. *coli* occurring as monomer [74]. The QMEAN score for quality estimation was 0.633 yielding in good model reliability.

Another novel immunogenic protein is NGO0777, named DNA-binding protein Hu, which is involved in the control of gene expression and other DNA transactions in bacteria [75]. Its homologue in *N*. *meningitidis* (NMB1230) is included in outer membrane preparations and outer membrane vaccines in two different studies [47,49]. NGO0777 was shown to be upregulated in a gonococcal deletion mutant lacking a *dam replacing gene* which is involved in DNA synthesis [76]. Furthermore, its homologue in *E*. *coli* is induced by treatment of cells with UV or mitomycin C [77]. Hence, it is involved in DNA repair in certain stress situations which could also be the case in cell adhesion or infection.

NGO1634 has been annotated as a hypothetical protein or putative phage associated protein, thus the knowledge about its expression, if any, and function is rather limited. Consequently, a real-time PCR analysis was performed to validate the presence of mRNA coding for this protein. It was shown that the gene encoding NGO1634 is transcribed into mRNA resulting in an expression of the according protein in culture under laboratory conditions. Nevertheless, no functional bacteriophages have been found in *N*. *gonorrhoeae* [4], although there are genomic regions that are related to lysogenic phage [78]. However, the region including NGO1634 is assumed to encode incomplete phage genomes. Hence, NGO1634 might be a protein of bacteriophage origin with a novel but yet unknown function in *N*. *gonorrhoeae*. BLAST results show a high identity of hits within *N*. *gonorrhoeae* strains, while only a few strains of other *Neisseria* species carry this gene. Future studies with monoclonal antibodies might reveal the precise localisation within the cell as well as the expression rate. Furthermore, a functional analysis of this protein might be achieved by generating deletion mutants to investigate the protein’s influence on infection, viability and gonococcal virulence.

NGO1796 is annotated as a ribosome recycling factor (rrf) and is involved in the recycling of ribosomes upon translation. It showed signal intensities in ELISA above fourfold of the cut-off of the negative control and had comparable signals to NGO0170, NGO0642 and NGO1634. Studies with an *E*. *coli* homologue showed that it is essential for bacterial growth and a possible target for novel antibacterial treatments [79].

In contrast, NGO0451, a replicative DNA helicase, showed signal intensities in the range of the negative control after full-length production despite promising signal intensities during phage library screening. A possible explanation is that the epitope is inaccessible to the antibody within the full-length protein, while the shorter oligopeptide does not suffer from these limitations. Furthermore, if conformational epitopes are formed and recognised, it is highly possible that the secondary structure of truncated oligopeptide and full-length protein differ. Consequently, it is essential to validate the immunogenic character on full-length protein level. Apart from these newly described immunogenic proteins, this method revealed several other proteins that have been described in literature before, yet mainly in the closely related pathogen *N*. *meningitidis* and were verified to be immunogenic in *N*. *gonorrhoeae* as full-length proteins within this study: NGO1043 [51], NGO1656 [48] and NGO1852 [46]. Proteins that are well-known to be immunogenic in several bacterial species as well as *N*. *meningitidis* were not produced in full-length. These proteins included NGO0592 [48], NGO0983 [80], NGO1429 [25,48], NGO2094 [81] and NGO2139 [82]. Furthermore, two proteins were identified that had been described as immunogenic in *N*. *gonorrhoeae* before: NGO1577, an outer membrane protein PIII [83] and NGO2095, a chaperonin 60 kDa subunit [84].

NGO1656 and NGO1657 were expressed as full-length proteins, since one selected clone contained DNA sequences of both proteins–the last 92 bp of NGO1657, a non-coding part of 48 bp and 131 bp of NGO1656. Notably, the non-coding part included three stop codons and the immunogenic character was only validated for downstream NGO1656. Packaging with Hyperphage leads to an enrichment of open reading frames (ORF) [40]. Inserts with stop codons and out-of-frame arrangements are largely removed as infectious phage particles are only obtained if the coded sequence of insert and gIII is expressed as a full-length fusion protein. Since the insert also contained a start codon and signal peptide of NGO1656 according to SignalP (http://www.cbs.dtu.dk/services/SignalP) it is most likely that only the sequence coding for NGO1656 was translated into a fusion protein with the phage coat protein pIII followed by periplasmic translocation in *E*. *coli* and phage assembly. Hence, the NGO1657 derived oligopeptide may not have been displayed on the selected phage clone and selection was due to the display of only NGO1656 explaining the full-length protein of NGO1657 to fail the proof of immunogenicity in ELISA. The homologue of NGO1656 in *N*. *meningitidis* (NMB0345) has been described to be immunogenic [53] and has been identified in outer membrane vesicles [49].

Several proteins were expressed as controls: NGO0564, a dihydrolipoamide acetyltransferase, was identified during phage library screening. It has been described as a potential candidate for a vaccine against *N*. *meningitidis* [46]. As expected it was strongly bound by a polyclonal anti-*N*. *gonorrhoeae* rabbit antibody in ELISA validating its immunogenic character for *N*. *gonorrhoeae* FA1090. NGO1043 is annotated as a hypothetical protein or lipoprotein in different homologues. The homologue NMB1468 was described as an immunogenic protein in *N*. *meningitidis* and its vaccine potential was evaluated as a promising vaccine candidate [51]. The immunogenic character of this protein was validated for *N*. *gonorrhoeae* in this work.

Two different proteins were chosen as negative controls. On the one hand, BB0069 (aminopeptidase II) of *Borrelia burgdorferi* was chosen as it is unrelated to any *N*. *gonorrhoeae* protein and contains only the recombinant protein and residual *E*. *coli* proteins that might be present after purification. On the other hand, NGO1500 was isolated after panning but the oligopeptide showed very low signal intensities during screenings. Nevertheless, it was selected after the third panning round indicating that there could possibly be an antibody binding event. The ELISA with the full-length protein revealed higher signal intensities than BB0069 which could be attributed to a low antibody titre. Hence, BB0069 was applied to calculate the ELISA cut-off.

Although all novel identified proteins are predicted to be localised in the cytoplasm except for NGO0170 (cytoplasmic membrane), future investigations are necessary to determine the exact localisation within the cell. Two of the identified oligopeptides—NGO1429 dnaK and NGO1852 RplL—with predicted cytoplasmic localisation were shown to be surface-exposed in *N*. *meningitidis* [85] or *N*. *gonorrhoeae* [86], respectively. Furthermore, several periplasmic ABC transporters comparable to NGO0170 were found to be surface-exposed in *N*. *meningitidis* [87,88]. Moreover, homologues of NGO0326, NGO0564, NGO0592, NGO0777, NGO0916, NGO0983, NGO1043, NGO2095 and NGO2139 were also detected in outer membrane fractions [49,88,89]. Whether the latter proteins were surface-exposed or remnants of vesicle production was not further analysed. However, in case of NGO0592 and NGO2095 the homologues NMB1313 and NMB1972 were confirmed to be surface-exposed [89]. Further experiments with specific antibodies could give insight into surface-exposure of proteins and gonococcal infection.

Three-dimensional structures of the six novel identified immunogenic proteins were predicted by I-TASSER and their closest PDB templates were compared with the results obtained by PSI-BLAST with three iterations. Templates of the obtained models were usually found around the top ten PSI-BLAST hits. After model prediction I-TASSER aligned the best model with all available PDB structures and identified the PDB structure with the highest similarity. This template was used for predicting the protein’s quaternary structure. In case of multimeric quaternary structures, identified oligopeptides were also mapped to those template models to predict their accessibility for antibodies. The best models were checked for their quality with the QMEAN server. All models, except for NGO1634, had QMEAN scores of around 0.6 indicating acceptable modelling accuracy for function inference. For NGO1634, PSI-BLAST could not identify good homologues, resulting in low reliability of I-TASSER models confirmed by a low QMEAN score of 0.34. However, this is due to a lack of homologous structures and proteins with homologous sequences. Hence, further investigation of NGO1634 could provide detailed information as to the protein’s potential function, localization and structure.

Oligopeptides that were identified during phage library screening were mapped to the protein models and gave further information of the localization of immunogenic parts of the protein. Since these regions elicited antibody binding they will be further investigated for epitope determination. Most likely, linear epitopes will be revealed, but conformational epitopes might have been formed within an oligopeptide structure presented on phage particles. Furthermore, virulence-associated proteins were identified such as NGO0326 and NGO1577 increasing the likelihood of discovering virulence-associated factors among the identified proteins. Particularly NGO1634 will be further examined. Additional analyses for further characterisation of these immunogenic proteins will be conducted. Careful attention will be put on epitope mapping. Moreover, additional studies will be necessary to confirm the immunogenic character of the identified proteins also in-vivo. Furthermore, recombinant antibodies against selected proteins will be generated to further assist in characterising the identified proteins. Finally, the identified proteins in this work might be suitable targets for the development of novel therapeutic measures against *N*. *gonorrrhoeae* and could provide crucial information for a better understanding of gonococcal infection.

## Materials and Methods

### Bacterial strains

For the isolation of genomic DNA the sequenced strain *N*. *gonorrhoeae* FA 1090 was grown on Columbia Blood Agar at 37°C under microaerophilic conditions for 48 h. 100 mL of Brain-Heart-Infusion broth (BHI) supplemented with 5% fetal calf serum (FCS) were inoculated with a single colony and grown for 24 h at 37°C under microaerophilic conditions and 140 rpm.

### Antibodies

A polyclonal rabbit antibody to *N*. *gonorrhoeae* (Acris BP1050 Lot# 2B04511) was used for the panning rounds and initial screenings. A goat anti-rabbit IgG (Fc-specific, Biomol 611–1103 Lot#29341) was used as capture antibody for the panning procedure. Three different antibodies against *N*. *gonorrhoeae* from separate immunisations and pools of individuals (Acris BP1050 Lot# 2B04511 and Lot# 7A03112; Abcam ab19962 Lot# GR204640-1) were deployed for the validation of full-length immunogenic proteins. A mouse anti-M13/fd/F1/pVIII antibody (Progen 61097 Lot# 8032501) was used for capturing of oligopeptide phage particles during the screening ELISA. A colorimetric readout for ELISA assays was achieved with a polyclonal goat secondary antibody to rabbit IgG conjugated to horseradish peroxidase (Abcam ab6721 Lot# GR3175-3).

### Construction of a *N*. *gonorrhoeae* genomic DNA library

The cells were harvested by centrifugation (3000 x g, 10 min) and the genomic DNA was isolated by phenol chloroform extraction or GeneJET Genomic DNA Purification Kit (Thermo Scientific K0721). 60 μg of DNA were fragmented by sonication upon extraction. The following protocol was repeated six times on ice: 2 min sonication with an amplitude of 126 μm/s^2^, cycle setting 5, followed by 2 min chilling on ice (settings according to Bandelin Sonopuls HD2200, sonotrode MS72). Subsequently, the DNA was concentrated using Amicon Ultra 0.5 mL centrifugal filters with a cut-off of 30 kDa. DNA fragments with sizes from 100 to 1000 bp were extracted from an agarose gel and the DNA ends were repaired with the Fast DNA End Repair Kit (Thermo Scientific) according to the manufacturer’s instructions. 700 ng of fragmented DNA were then ligated into the *Pme*I digested pHORF3 vector (1000 ng) [41], followed by three transformations per ligation into *E*. *coli* TOP10F’ (Invitrogen) by electroporation. The number of required clones to cover the genome was calculated with the following equation [90]:

$$N=\text{ln}(1-P)/\text{ln}(1-\frac{a}{b})$$ (1)

N = number of required clones

P = probability that a certain fragment is present (set to 99.9%)

a = average size of the DNA inserts (depending on library)

b = total size of the *N*. *gonorrhoeae* FA 1090 genome (2,153,922 bp)

### Packaging of a phage display library with Hyperphage

The library was packaged using Hyperphage [38,39] as described before with some alterations [40,41]. By packaging the genomic DNA library with Hyperphage, ORFs are enriched and the resulting oligopeptides are presented on the phage particles for panning. 400 mL 2x YT-GA (2x yeast-tryptone broth supplemented with 0.1 M glucose and 100 μg/mL ampicillin) were inoculated with a library stock to an OD_600_ < 0.1. Following infection, the pellet was resuspended in 400 mL 2x YT-AK (2x YT containing 100 μg/mL ampicillin and 50 μg/mL kanamycin) and phage particles produced at 30°C and 250 rpm over-night. Cells were then centrifuged for 20 min at 10,000 x g and phage particles in the supernatant were precipitated with 1/5 volume 20% (w/v) polyethylene glycol (PEG6000)/2.5 M NaCl solution for 3 h on ice with gentle shaking. Phage particles were then pelleted for 1 h at 10,000 x g and resuspended in 10 mL phage dilution buffer (10 mM TrisHCl pH 7.5, 20 mM NaCl, 2 mM EDTA). Remaining bacteria were pelleted by an additional centrifugation step of 10 min at 20,000 x g and subsequently, the solution was filtered through a 0.45 μm filter. The filtrate was again precipitated with 1/5 PEG/NaCl for 1 h and subsequent centrifugation for 30 min at 20,000 x g. Pellets were resuspended in 1 mL phage dilution buffer and residual bacterial cells removed by centrifugation for 1 min at 16.000 x g. The final supernatant containing the oligopeptide presenting phages was stored at 4°C. Phage titres were determined by titration [91].

### Library validation by Colony PCR and Sequencing

To check the library quality a number of random *E*. *coli* colonies were analysed by colony PCR and sequenced before and after packaging with Hyperphage. Therefore, the primers MHLacZPro_f (5'-GGCTCGTATGTTGTGTGG-3') and MHgIII_r (5'-GGAAAGACGACAAAACTTTAG-3') were used with the following PCR protocol: 98°C 30 s, 98°C 10 s, 56°C 20 s (35 cycles), 72°C 60 s and a final extension of 72°C for 600 s. The DNA was separated and analysed by capillary electrophoresis (Qiaxcel Advanced). Additionally, plasmid DNA was sequenced with the primers used for colony PCR to verify the correct inserts and the ORF enrichment after packaging.

### Selection of immunogenic oligopeptide phage by panning

Two wells of a Maxisorp W 96 well microplate were coated with 150 μL of a goat anti-rabbit IgG (Fc-specific) antibody (5 μg/mL) in phosphate buffered saline (PBS) at 4°C overnight. Subsequently, the wells were washed thrice with PBS supplemented with 0.1% Tween20 (PBST) and blocked for 1 h with PBST supplemented with 2% (w/v) milk powder (2% MPBST). For each panning well, two wells of a Maxi-Sorp W 96 well plate (Nunc) were coated with 200 μL 5 x 10^10^ cfu Hyperphage in PBS at 4°C overnight and blocked with 2% MPBST for 1 h. The polyclonal rabbit antibody to *N*. *gonorrhoeae* was diluted 1:200 in 2% MPBST and preincubated twice in the Hyperphage coated wells for 1 h to eliminate IgGs binding to helperphage. After preincubation, the polyclonal antibody was incubated for 1.5 h in the wells coated with the goat anti-rabbit IgG antibody. 150 μL of the *N*. *gonorrhoeae* phage library (corresponding to 1.1 x 10^10^ cfu oligopeptide phage particles of the phage library) plus 50 μL MPBST were then incubated in the wells with the captured anti-*N*. *gonorrhoeae* antibodies for 2 h. 150 μL of amplified phage of the previous panning round were used for the second and third round. Unbound and phages with low affinities were rinsed out by ten stringent washing steps. Each panning round the washing steps were increased by ten with a total number of three panning rounds. After washing, bound phage particles were eluted by trypsination with 200 μL of 10 μg/mL trypsin in PBS for 30 min at 37°C. Eluted phages of both wells were combined and 10 μL were used for titration. The remaining 390 μL were used to infect 20 mL of an *E*. *coli* TOP10F’ culture grown to an OD_600_ of 0.5. The cells were incubated for 30 min at 37°C and harvested by centrifugation for 10 min at 3250 x g. The pellet was resuspended in 250 μL 2x YT-GA. The bacterial suspension was plated onto 15 cm 2x YT-GA agar plates and incubated overnight at 37°C. Colonies were swept off with 5 mL 2x YT-GA medium, then 50 mL of 2x YT-GA medium were inoculated with the bacterial suspension to an OD_600_ of < 0.1 and grown to an OD_600_ of 0.5 at 37°C and 250 rpm. 5 mL of the bacterial culture (approx. 2.5 x 10^9^ cells) were infected in a ratio of 1:20 resulting in 5 x 10^10^ cfu Hyperphage. The suspension was incubated at 37°C for 30 min without shaking and another 30 min at 37°C and 250 rpm. To dispose the glucose that inhibits phage expression, the infected cells were harvested by centrifugation for 10 min at 3250 x g. The remaining pellet was resuspended in 30 mL 2x YT-AK and incubated at 30°C and 250 rpm overnight for phage production. The bacterial cells were then pelleted by centrifugation for 20 min at 3250 x g and the remaining supernatant was used for phage particle precipitation with PEG/NaCl (20% (w/v) PEG 6000, 2.5 M NaCl). 30 mL of supernatant were incubated for 1 h with 6 mL PEG/NaCl on ice with slight shaking on a rocker, followed by centrifugation at 6000 x g for 1 h at 4°C. The phage pellet was resuspended in 500 μL phage dilution buffer (10 mM TrisHCl pH 7.5, 20 mM NaCl, 2 mM EDTA), centrifuged in a microcentrifuge at 16,100 x g for 1 min and the supernatant was used for further panning rounds. Eluted phage particles from the third panning round were not further amplified and used for titration. Single colonies were then used for single oligopeptide phage production.

### Production of single oligopeptide phage clones for screening

Single oligopeptide phage clones were produced by inoculating 175 μL 2x YT-GA medium each with a single colony from the titration plate in a polypropylene 96 well U bottom plate (Greiner bio-one). The cultures were incubated at 37°C and 800 rpm shaking overnight. 165 μL 2x YT-GA medium per well were inoculated with 10 μL of the overnight cultures and incubated at 37°C and 800 rpm for 2 h. Subsequently, the bacteria were infected with 5 x 10^9^ cfu Hyperphage and incubated for 30 min at 37°C without shaking and 30 min at 37°C and 800 rpm. The bacteria were pelleted by centrifugation at 3250 x g for 10 min and the pellets were resuspended in 175 μL/well 2x YT-AK and incubated overnight at 30°C and 800 rpm. The produced phage in the supernatant were transferred to another plate and precipitated with 1/5 volume of PEG/NaCl solution for 1 h at 4°C. Next, precipitated phage particles were pelleted by centrifugation at 3250 x g for 1 h and the pellets dissolved in 150 μL PBS. Remaining bacterial cells were separated by another centrifugation step and the phage containing supernatants stored in a new plate at 4°C and used for screening ELISA.

### ELISA screening of oligopeptide phage clones

Oligopeptide phage particles were captured by a monoclonal mouse anti-M13 (B62-FE2, Progen) antibody for screening. 100 μL of 250 ng/mL antibody in PBS were coated over-night at 4°C and subsequently blocked with 2% MPBST. The wells were washed with 200 μL PBST thrice after each incubation step. 150 μL of the monoclonal phage clones were incubated for 2 h. A polyclonal rabbit antibody against *N*. *gonorrhoeae* was diluted 1:1000 in 2% MPBST supplemented with 10% *E*. *coli* TOP10F’ lysate and 1 x 10^10^ cfu/mL Hyperphage per mL of the dilution and preincubated for 2 h. Then, the preincubated antibody was incubated on the captured phage particles for 2 h and detected by a goat anti-rabbit IgG antibody conjugated to horseradish peroxidase (HRP) (1:20,000) for 1.5 h. Visualisation was achieved by adding 100 μL TMB (3,3’,5,5’-tetramethylbenzidine) solution and the reaction was stopped by 100 μL 1 N sulphuric acid. The absorbance of yellow colour change was measured at 450 nm using a FLUOstar Omega microplate reader (BMG Labtech).

### Cloning of full size genes

For the validation of immunogenicity of full-length proteins genes were amplified from genomic DNA by PCR using primers listed in Table 2. The gene of the negative control BB0069 was amplified with genomic DNA from *Borrelia burgdorferi* B31. Phusion Polymerase (Thermo Scientific F-530L) was used for amplification of full-length genes according to following protocol: 98°C 30 s, 98°C 10 s, annealing temperature primer dependent 20 s, 72°C 20 s, 30 cycles, 72°C 10 min. The amplified genes were digested with two restriction enzymes in each case: *Nde*I and *Not*I for pET21A+ or *Nhe*I and *Not*I if the gene carried a signal peptide or a restriction site for one of the enzymes. Corresponding bands were cut out from a 1% agarose gel, purified by NucleoSpin Gel and PCR Clean-Up (Macherey-Nagel 740609.250) and used for cloning into the appropriately digested vector pET21A+ (*Nde*I/*Not*I) or pET21A+pelB (*Nhe*I/*Not*I). *E*. *coli* BLR-DE3 cells were transformed with the ligated plasmids. Finally, positive clones were identified by colony PCR using the oligonucleotide primers pet21a_CPCR3_for (5´-GAGCGGATAACAATTCCCC-3´) and pet21a_CPCR3_rev (5´-GCAGCCAACTCAGCTTCC-3´) and by sequencing.

**Table 2:** Primers utilized for full-length gene cloning.

Locus Tag	Primer name	Primer sequence (5’—3’)
NGO0170	Ngon_0170_for	AGATATACATATGGGGTGTTTACCTTTG
	Ngon_0170_rev	AGAGCGTGCGGCCGC**A**TATGAGGCGCACCAGT
NGO0326	Ngon_0326_for	AGATATACATATGACAGCTAAAGGACAAATGTTGCA
	Ngon_0326_rev	CTCGAGTGCGGCCGC**A**TATTCGGCAGGCTGCTGGACG
NGO0451	Ngon_0451_for	AAGGAGATATACATATGAACGATTACGCAGCTATGCCG
	Ngon_0451_rev	CTCGAGTGCGGCCGC**A**TAATCTTCTATCTTTGCCTCCTCGG
NGO0564	Ngon_0546_for	AAGGAGATATACATATGAGTATCGTAGAAATCAAAGTCCC
	Ngon_0546_rev	GACGTGTGCGGCCGC**A**TATAAGGTAATGCGGCGGAAGT
NGO0642	Ngon_0642_for	AAGGAGATATAGCTAGCATGACCAATAAACCCGCCAAACGCC
	Ngon_0642_rev	GTCGAGTGCGGCCGC**C**CAGGCGGAGGATGCCGCCG
NGO0777	Ngon_0777_for	AAGGAGATATACATATGAACAAGTCTGAATTGATCG
	Ngon_0777_rev	CTCGAGTGCGGCCGC**A**TACAGTGCGTCTTTCAATG
NGO0916	Ngon_0916_for	AAGGAGATATACATATGATTATTGATGTAAAAGTACCTATGCTG
	Ngon_0916_rev	CTCGAGTGCGGCCGC**A**TACAGATCCAACAACAGGC
NGO1043	Ngon_1043_for	AAGGAGATATAGCTAGCAAACAGGAGGTTAAAGAAGCGGC
	Ngon_1043_rev	CTCGAGTGCGGCCGC**A**TATTTGGCGGCGTCTTTCAT
NGO1500	Ngon_1500_for	AGATATACATATGGAAAATATCGGCAGGCAGC
	Ngon_1500_rev	TCGAGTGCGGCCGC**A**TAACCCAAAGACACCATTTCAATCTG
NGO1634	Ngon_1634_for	AAGGAGATATACATATGAGTGCGAGGCTGATGGG
	Ngon_1634_rev	CACGAGTGCGGCCGC**A**CAAAATCTGATGGTTCTTAGCGCAA
NGO1656	Ngon_1656_for	AAGGAGATATACATATGAAAGCAAAAATCCTGACTTCCG
	Ngon_1656_rev	CTCGAGTGCGGCCGC**A**TATTTTGCAGGTTTGATGTTTGCC
NGO1657	Ngon_1657_for	AAGGAGATATACATATGCCGGCTGTCGATTTGATC
	Ngon_1657_rev	CTCGAGTGCGGCCGC**A**CAGTCCGCCGTATGGAAATACT
NGO1796	Ngon_1796_for	AAGGAGATATACATATGATCAACGATATTCAAAAAACAGC
	Ngon_1796_rev	CTCGAGTGCGGCCGC**A**TAAACCGCCATCAGGTCTTC
NGO1852	Ngon_1852_for	AAGGAGATATACATATGGCTATTACTAAAGAAGACATT
	Ngon_1852_rev	CTCGAGTGCGGCCGC**A**TATTTGATTTCGACTTTAGCG
BB0069	BB0069_for	AAGGAGATATACATATGGAGAAAGATTTAATAAAATA
	BB0069_rev	ATCGAGTGCGGCCGC**A**TATATTGCGAATTTACCAG

Restriction sites are underlined, modified nucleotides for stop codon removal are marked bold, annealing temperatures were calculated for Phusion polymerase with a Tm calculator (life Technologies).

### Production and purification of full-length proteins

200 mL 2x YT-GA broth were inoculated with 10 mL overnight culture and cultivated in a baffled flask to an OD_600_ of 0.6 at 37°C and 120 rpm. Expression was induced with a final concentration of 1 mM IPTG for 5 h with a decreased temperature of 30°C, followed by centrifugation at 3000 x g for 20 min for cell harvesting. Cells were lysed and all proteins purified under denaturing conditions by resuspending the cell pellet in His-tag binding buffer pH 8 (50 mM Na_2_HPO_4_, 100 mM NaCl, 10 mM imidazol, 8 M urea) and incubation for 1 h by over-head rotation, followed by sonication (6 cycles of 10 s 50% power, 10 s incubation on ice, Sonotrode MS72, Bandelin). Subsequently 0.5 mL Ni-NTA agarose slurry (Qiagen 30210) was added to the disrupted cell solution and incubated for 2 h by over-head rotation. Then, the solution was loaded onto a polypropylene column. The agarose settled by gravity flow and was washed with 10 mM, 30 mM and 50 mM imidazole (50 mM Na_2_HPO_4_, 300 mM NaCl, 8 M urea, pH 8). Elution was achieved with 3 x 1.25 mL PBS pH 7.4 supplemented with 100 mM EDTA and 8 M urea.

### SDS-PAGE

Full-length proteins were analysed by 12%–15% SDS-PAGE depending on expected protein size. Gels were cast according to the protocol of Sambrook and Russell [26]. 0.5 μg of protein sample was mixed with 5x Lane Marker Reducing Sample Buffer (Thermo Scientific 39000) and boiled at 95°C for 5 min. PageRuler Plus Prestained Ladder (Thermo Scientific 26619) and Spectra Multicolor Low Range Protein Ladder (Thermo Scientific 26628) were used as size marker. The samples were stacked for 10 min at 60 V, followed by separation for 60 min at 110 V. The gels were then stained with PageBlue Protein Staining Solution (Thermo Scientific 24620).

### ELISA for validation of immunogenicity of the full-length proteins

1 μg of protein was coated to a 96 well microplate (MaxiSorp, Nunc) in 50 mM NaHCO_3_ pH 9.6 at 4°C overnight. Blocking was performed with 2% MPBST for 1.5 h. Polyclonal rabbit antibodies against *N*. *gonorrhoeae* were diluted 1:1000 in 2% MPBST supplemented with 10% *E*. *coli* lysate and incubated for 1.5 h. After each incubation step the wells were washed thrice with 200 μL PBST. The wells were then incubated at RT with the preincubated antibody for 2 h. Bound rabbit IgGs were detected with goat anti-rabbit IgG antibody conjugated to HRP (1:20,000). Visualisation was achieved by adding 100 μL TMB solution. The reaction was stopped by adding 100 μL 1 N sulphuric acid. Subsequently, the absorbance was measured at 450 nm using a FLUOstar Omega microplate reader (BMG Labtech).

### Isolation of total RNA and transcription of first strand cDNA

For the isolation of total RNA a biological triplicate each consisting of a 10 mL *N*. *gonorrhoeae* culture were used and RNA was isolated by using RNAprotect Bacteria Reagent (Qiagen 76506) and RNeasy Mini Kit (Qiagen 74104) following the manufacturer’s instructions. Additionally to the on-column DNA digestion, a second DNA digestion was used to eliminate residual DNA contaminations. The RNA Integrity was checked with an Agilent 2100 Bioanalyzer using the RNA 6000 Pico Kit (Agilent 5067–1513). The RNA integrity number (RIN) lies within the range of 0 to 10. Higher numbers indicate an intact RNA while low numbers refer to degraded RNA. First strand cDNA was generated with RevertAid First Strand cDNA Synthesis Kit (Thermo Scientific K1622) according to the manufacturer’s instructions using random hexamer primers. Simultaneously, a negative control with no reverse transcriptase (RT-) and a no template control (NTC) were carried out.

### Real-time PCR

The expression of NGO1634 (annotated as hypothetical protein, putative phage associated) was verified by real-time PCR with the three reverse transcribed cDNAs as biological triplicate. As negative controls a no template control for the PCR reaction and the two negative controls from the reverse transcription were included. A part of the gene of NGO0715 (glucose-6-phosphate 1-dehydrogenase) was applied as positive control. The reaction was set-up with Maxima SYBR Green qPCR Master Mix (2x) (Thermo Scientific K0251) following the manufacturer’s instructions with first strand cDNA as template. The real-time PCR was run on a Roche LightCycler 2.0 using the following program: 95°C 10 min, 40 cycles of 95°C 15 s, 50°C 30 s, 72°C 30 s with a single data acquisition after the elongation step. Additionally, a melting curve was recorded starting at 95°C with 0.05°C/s slope decreasing to 45°C and continuous fluorescence acquisition (data not shown). The primers which were used for real-time PCR are listed in Table 3.

**Table 3:** Primers utilized for real-time PCR.

Locus Tag	Primer name	Primer sequence (5’—3’)
NGO1634	N_gon_1634_real_fw	CGTACGGCAGCATATCAAGT
	N_gon_1634_real_rv	ACGTTGATGCGGTAGATGTC
NGO0715	N_gon_gdh_real_fw	GCATGAACGGCTATCTTGAA
	N_gon_gdh_real_rv	GGTACGCAGGTAGAAGGGAA

NGO0715 (glucose-6-phosphate 1-dehydrogenase) was applied as positive control.

### Bioinformatics

Primers were manually designed and checked with PCR Primer Stats from the Sequence Manipulation Suite [92]. Primers for qPCR were designed with GeneScript Real-Time PCR (TaqMan) Primer Design. Sequenced inserts were identified by BLAST [93]. Localisation prediction was performed with PSORTb v3.0.2 [94]. Signal peptides were predicted using SignalP 4.1 [95]. 3-dimensional structures were predicted using I-TASSER [60]. All models were selected based upon their C-score, which is a confidence score used by I-TASSER to estimate the quality of the predicted models. The C-score is usually in a range between -5 and 2. A C-score with a higher value represents a model with a high confidence and vice-versa. Models were visualized using Chimera [96].Model reliability was additionally checked by QMEAN [64]. A summary of available homologue structures was obtained by running PSI-BLAST (3 iterations) against the PDB database (S1 Table). Evaluation of results was performed by Microsoft Excel and OriginPro 9.1 G (Originlab).

## Supporting Information

S1 Fig Schematic view of the protein primary sequence (yellow bar) from protein NGO0592 with annotated identified oligopeptides (brown bars). In total, three oligopeptides were identified; two of them overlapping between amino acids 79 and 148, the third in a different part of the protein spanning from amino acid residue 240 to 285.
(TIF)

S2 Fig Amplification curves of the real-time PCR (n = 3). The Cp values were 27.25 ± 0.82 for NGO1634, 20.94 ± 1.7 for GDH (NGO0715) and > 35 for the controls.
(TIF)

S1 Table Summary of available homologous structures of all novel immunogenic proteins. Query coverage, E-values and sequence similarities obtained by PSI-BLAST (3 iterations) against the PDB database are listed. Only an excerpt of the complete list is shown containing the top hits and the template chosen by I-TASSER (highlighted in grey). For NGO0170 the homologue was hit number 76; PDB hits 11–75 are not included.
(XLSX)

## References

[1] WHO. WHO | Global action plan to control the spread and impact of antimicrobial resistance in *Neisseria gonorrhoeae* In: WHO [Internet]. 2012 Available: http://www.who.int/reproductivehealth/publications/rtis/9789241503501/en/, Accessed 9 April 2015.

[2] Svensson L, Weström L, Ripa KT, Mårdh PA. Differences in some clinical and laboratory parameters in acute salpingitis related to culture and serologic findings. Am J Obstet Gynecol. 1980;138: 1017–1021. 6451176 10.1016/0002-9378(80)91099-6

[3] Rotman E, Seifert HS. The genetics of Neisseria species. Annu Rev Genet. 2014;48: 405–431. 10.1146/annurev-genet-120213-092007 25251852

[4] Bennett JE, Dolin R, Blaser MJ. Mandell, Douglas, and Bennett’s Principles and Practice of Infectious Diseases. Elsevier Health Sciences; 2014.

[5] Update to CDC’s Sexually Transmitted Diseases Treatment Guidelines, 2010: Oral Cephalosporins No Longer a Recommended Treatment for Gonococcal Infections [Internet].]. Available: http://www.cdc.gov/mmwr/preview/mmwrhtml/mm6131a3.htm. Accessed 9 April 2015. 22874837

[6] Tapsall JW, Ndowa F, Lewis DA, Unemo M. Meeting the public health challenge of multidrug- and extensively drug-resistant Neisseria gonorrhoeae. Expert Rev Anti Infect Ther. 2009;7: 821–834. 10.1586/eri.09.63 19735224

[7] Wallin J. Gonorrhoea in 1972. A 1-year study of patients attending the VD Unit in Uppsala. Br J Vener Dis. 1975;51: 41–47. 1125748 10.1136/sti.51.1.41 PMC1045109

[8] Handsfield HH, Lipman TO, Harnisch JP, Tronca E, Holmes KK. Asymptomatic Gonorrhea in Men. N Engl J Med. 1974;290: 117–123. 10.1056/NEJM197401172900301 4202519

[9] John J, Donald WH. Asymptomatic urethral gonorrhoea in men. Br J Vener Dis. 1978;54: 322–323. 709346 10.1136/sti.54.5.322 PMC1045531

[10] Holmes K. Sexually Transmitted Diseases, Fourth Edition McGraw-Hill Education; 2008.

[11] Workowski KA, Berman SM, Douglas J John M. Emerging Antimicrobial Resistance in Neisseria gonorrhoeae: Urgent Need to Strengthen Prevention Strategies. Ann Intern Med. 2008;148: 606–613. 10.7326/0003-4819-148-8-200804150-00005 18413622

[12] Unemo M, Nicholas RA. Emergence of multidrug-resistant, extensively drug-resistant and untreatable gonorrhea. Future Microbiol. 2012;7: 1401–1422. 10.2217/fmb.12.117 23231489 PMC3629839

[13] Ohnishi M, Golparian D, Shimuta K, Saika T, Hoshina S, Iwasaku K, et al Is Neisseria gonorrhoeae Initiating a Future Era of Untreatable Gonorrhea?: Detailed Characterization of the First Strain with High-Level Resistance to Ceftriaxone. Antimicrob Agents Chemother. 2011;55: 3538–3545. 10.1128/AAC.00325-11 21576437 PMC3122416

[14] Cole MJ, Spiteri G, Chisholm SA, Hoffmann S, Ison CA, Unemo M, et al Emerging cephalosporin and multidrug-resistant gonorrhoea in Europe. Euro Surveill Bull Eur Sur Mal Transm Eur Commun Dis Bull. 2014;19: 20955. 10.2807/1560-7917.es2014.19.45.20955 25411689

[15] Barbee LA, Dombrowski JC. Control of Neisseria Gonorrhoeae in the Era of Evolving Antimicrobial Resistance. Infect Dis Clin North Am. 2013;27 10.1016/j.idc.2013.08.001 PMC3843156 24275266

[16] Hagblom P, Segal E, Billyard E, So M. Intragenic recombination leads to pilus antigenic variation in Neisseria gonorrhoeae. Nature. 1985;315: 156–158. 2859529 10.1038/315156a0

[17] Stern A, Brown M, Nickel P, Meyer TF. Opacity genes in Neisseria gonorrhoeae: control of phase and antigenic variation. Cell. 1986;47: 61–71. 3093085 10.1016/0092-8674(86)90366-1

[18] Mulks MH, Plaut AG. IgA protease production as a characteristic distinguishing pathogenic from harmless neisseriaceae. N Engl J Med. 1978;299: 973–976. 10.1056/NEJM197811022991802 99655

[19] Pohlner J, Halter R, Beyreuther K, Meyer TF. Gene structure and extracellular secretion of Neisseria gonorrhoeae IgA protease. Nature. 1987;325: 458–462. 10.1038/325458a0 3027577

[20] Papp JR, Schachter J, Gaydos CA, Van Der Pol B. Recommendations for the Laboratory-Based Detection of Chlamydia trachomatis and Neisseria gonorrhoeae—2014. MMWR Recomm Rep Morb Mortal Wkly Rep Recomm Rep Cent Dis Control. 2014;63: 1–19. PMC4047970 24622331

[21] Workowski KA, Berman S, Centers for Disease Control and Prevention (CDC). Sexually transmitted diseases treatment guidelines, 2010. MMWR Recomm Rep Morb Mortal Wkly Rep Recomm Rep Cent Dis Control. 2010;59: 1–110. 21160459

[22] LaFrentz BR, LaPatra SE, Call DR, Wiens GD, Cain KD. Identification of immunogenic proteins within distinct molecular mass fractions of Flavobacterium psychrophilum. J Fish Dis. 2011;34: 823–830. 10.1111/j.1365-2761.2011.01297.x 21988354

[23] Nowalk AJ, Gilmore RD, Carroll JA. Serologic Proteome Analysis of Borrelia burgdorferi Membrane-Associated Proteins. Infect Immun. 2006;74: 3864–3873. 10.1128/IAI.00189-06 16790758 PMC1489744

[24] Burlak C, Hammer CH, Robinson M-A, Whitney AR, McGavin MJ, Kreiswirth BN, et al Global analysis of community-associated methicillin-resistant Staphylococcus aureus exoproteins reveals molecules produced in vitro and during infection. Cell Microbiol. 2007;9: 1172–1190. 10.1111/j.1462-5822.2006.00858.x 17217429 PMC2064037

[25] Mendum TA, Newcombe J, McNeilly CL, McFadden J. Towards the Immunoproteome of Neisseria meningitidis. PLoS ONE. 2009;4 10.1371/journal.pone.0005940 PMC2691954 19529772

[26] Sambrook J, Russell DW. Molecular Cloning: A Laboratory Manual. CSHL Press; 2001.

[27] Zhu H, Hu S, Jona G, Zhu X, Kreiswirth N, Willey BM, et al Severe acute respiratory syndrome diagnostics using a coronavirus protein microarray. Proc Natl Acad Sci U S A. 2006;103: 4011–4016. 10.1073/pnas.0510921103 16537477 PMC1449637

[28] Hoppe S, Bier FF, von Nickisch-Rosenegk M, Nickisch-Rosenegk MV. Rapid identification of novel immunodominant proteins and characterization of a specific linear epitope of Campylobacter jejuni. PloS One. 2013;8: e65837 10.1371/journal.pone.0065837 23734261 PMC3667084

[29] Danckert L, Hoppe S, Bier FF, von Nickisch-Rosenegk M. Rapid identification of novel antigens of Salmonella Enteritidis by microarray-based immunoscreening. Mikrochim Acta. 2014;181: 1707–1714. 10.1007/s00604-014-1197-6 25253911 PMC4167438

[30] Beranova-Giorgianni S. Proteome analysis by two-dimensional gel electrophoresis and mass spectrometry: strengths and limitations. TrAC Trends Anal Chem. 2003;22: 273–281. 10.1016/S0165-9936(03)00508-9

[31] Rabilloud T, Chevallet M, Luche S, Lelong C. Two-dimensional gel electrophoresis in proteomics: Past, present and future. J Proteomics. 2010;73: 2064–2077. 10.1016/j.jprot.2010.05.016 20685252

[32] Urquhart BL, Cordwell SJ, Humphery-Smith I. Comparison of predicted and observed properties of proteins encoded in the genome of Mycobacterium tuberculosis H37Rv. Biochem Biophys Res Commun. 1998;253: 70–79. 10.1006/bbrc.1998.9709 9875222

[33] Smith GP. Filamentous fusion phage: novel expression vectors that display cloned antigens on the virion surface. Science. 1985;228: 1315–1317. 4001944 10.1126/science.4001944

[34] Hoogenboom HR, Winter G. By-passing immunisation. Human antibodies from synthetic repertoires of germline VH gene segments rearranged in vitro. J Mol Biol. 1992;227: 381–388. 1404359 10.1016/0022-2836(92)90894-p

[35] Dübel S, Stoevesandt O, Taussig MJ, Hust M. Generating recombinant antibodies to the complete human proteome. Trends Biotechnol. 2010;28: 333–339. 10.1016/j.tibtech.2010.05.001 20538360

[36] Hoogenboom HR. Selecting and screening recombinant antibody libraries. Nat Biotechnol. 2005;23: 1105–1116. 10.1038/nbt1126 16151404

[37] Kügler J, Zantow J, Meyer T, Hust M. Oligopeptide m13 phage display in pathogen research. Viruses. 2013;5: 2531–2545. 10.3390/v5102531 24136040 PMC3814601

[38] Rondot S, Koch J, Breitling F, Dubel S. A helper phage to improve single-chain antibody presentation in phage display. Nat Biotechnol. 2001;19: 75–78. 10.1038/83567 11135557

[39] Soltes G, Hust M, Ng KKY, Bansal A, Field J, Stewart DIH, et al On the influence of vector design on antibody phage display. J Biotechnol. 2007;127: 626–637. 10.1016/j.jbiotec.2006.08.015 16996161 PMC1866265

[40] Hust M, Meysing M, Schirrmann T, Selke M, Meens J, Gerlach G-F, et al Enrichment of open reading frames presented on bacteriophage M13 using hyperphage. BioTechniques. 2006;41: 335–342. 16989094 10.2144/000112225

[41] Kügler J, Nieswandt S, Gerlach GF, Meens J, Schirrmann T, Hust M. Identification of immunogenic polypeptides from a Mycoplasma hyopneumoniae genome library by phage display. Appl Microbiol Biotechnol. 2008;80: 447–458. 10.1007/s00253-008-1576-1 18636254

[42] Naseem S, Meens J, Jores J, Heller M, Dübel S, Hust M, et al Phage display-based identification and potential diagnostic application of novel antigens from Mycoplasma mycoides subsp. mycoides small colony type. Vet Microbiol. 2010;142: 285–292. 10.1016/j.vetmic.2009.09.071 19900769

[43] Meyer T, Schirrmann T, Frenzel A, Miethe S, Stratmann-Selke J, Gerlach GF, et al Identification of immunogenic proteins and generation of antibodies against Salmonella Typhimurium using phage display. BMC Biotechnol. 2012;12: 29 10.1186/1472-6750-12-29 22703709 PMC3423037

[44] Becker M, Felsberger A, Frenzel A, Shattuck WMC, Dyer M, Kügler J, et al Application of M13 phage display for identifying immunogenic proteins from tick (Ixodes scapularis) saliva. BMC Biotechnol. 2015;15: 43 10.1186/s12896-015-0167-3 26024663 PMC4449557

[45] Türeci O, Sahin U, Pfreundschuh M. Serological analysis of human tumor antigens: molecular definition and implications. Mol Med Today. 1997;3: 342–349. 9269687 10.1016/s1357-4310(97)01081-2

[46] Ala’Aldeen D a. A, Westphal AH, Kok AD, Weston V, Atta MS, Baldwin TJ, et al Cloning, sequencing, characterisation and implications for vaccine design of the novel dihydrolipoyl acetyltransferase of Neisseria meningitidis. J Med Microbiol. 1996;45: 419–432. 10.1099/00222615-45-6-419 8958245

[47] Gil J, Betancourt LZH, Sardiñas G, Yero D, Niebla O, Delgado M, et al Proteomic study via a non-gel based approach of meningococcal outer membrane vesicle vaccine obtained from strain CU385: a road map for discovering new antigens. Hum Vaccin. 2009;5: 347–356. 19377283 10.4161/hv.5.5.7367

[48] Williams JN, Skipp PJ, O’Connor CD, Christodoulides M, Heckels JE. Immunoproteomic Analysis of the Development of Natural Immunity in Subjects Colonized by Neisseria meningitidis Reveals Potential Vaccine Candidates. Infect Immun. 2009;77: 5080–5089. 10.1128/IAI.00701-09 19737898 PMC2772525

[49] Vaughan TE, Skipp PJ, O’Connor CD, Hudson MJ, Vipond R, Elmore MJ, et al Proteomic analysis of Neisseria lactamica and Neisseria meningitidis outer membrane vesicle vaccine antigens. Vaccine. 2006;24: 5277–5293. 10.1016/j.vaccine.2006.03.013 16682101

[50] Black JR, Black WJ, Cannon JG. Neisserial antigen H.8 is immunogenic in patients with disseminated gonococcal and meningococcal infections. J Infect Dis. 1985;151: 650–657. 3919117 10.1093/infdis/151.4.650

[51] Hsu C-A, Lin W-R, Li J-C, Liu Y-L, Tseng Y-T, Chang C-M, et al Immunoproteomic identification of the hypothetical protein NMB1468 as a novel lipoprotein ubiquitous in Neisseria meningitidis with vaccine potential. PROTEOMICS. 2008;8: 2115–2125. 10.1002/pmic.200700574 18491322

[52] Kurz S, Hübner C, Aepinus C, Theiss S, Guckenberger M, Panzner U, et al Transcriptome-based antigen identification for Neisseria meningitidis. Vaccine. 2003;21: 768–775. 10.1016/S0264-410X(02)00596-0 12531357

[53] Williams JN, Weynants V, Poolman JT, Heckels JE, Christodoulides M. Immuno-proteomic analysis of human immune responses to experimental Neisseria meningitidis outer membrane vesicle vaccines identifies potential cross-reactive antigens. Vaccine. 2014;32: 1280–1286. 10.1016/j.vaccine.2013.12.070 24486354

[54] Hubálek M, Hernychová L, Havlasová J, Kasalová I, Neubauerová V, Stulík J, et al Towards proteome database of Francisella tularensis. J Chromatogr B. 2003;787: 149–177. 10.1016/S1570-0232(02)00730-4 12659739

[55] Qamra R, Mande SC, Coates ARM, Henderson B. The unusual chaperonins of Mycobacterium tuberculosis. Tuberculosis. 2005;85: 385–394. 10.1016/j.tube.2005.08.014 16253564

[56] Pizza M, Scarlato V, Masignani V, Giuliani MM, Aricò B, Comanducci M, et al Identification of Vaccine Candidates Against Serogroup B Meningococcus by Whole-Genome Sequencing. Science. 2000;287: 1816–1820. 10.1126/science.287.5459.1816 10710308

[57] Tsolakos N, Brookes C, Taylor S, Gorringe A, Tang CM, Feavers IM, et al Identification of vaccine antigens using integrated proteomic analyses of surface immunogens from serogroup B Neisseria meningitidis. J Proteomics. 2014;101: 63–76. 10.1016/j.jprot.2014.02.013 24561796

[58] Yang J, Yan R, Roy A, Xu D, Poisson J, Zhang Y. The I-TASSER Suite: protein structure and function prediction. Nat Methods. 2015;12: 7–8. 10.1038/nmeth.3213 25549265 PMC4428668

[59] Roy A, Kucukural A, Zhang Y. I-TASSER: a unified platform for automated protein structure and function prediction. Nat Protoc. 2010;5: 725–738. 10.1038/nprot.2010.5 20360767 PMC2849174

[60] Zhang Y. I-TASSER server for protein 3D structure prediction. BMC Bioinformatics. 2008;9: 40 10.1186/1471-2105-9-40 18215316 PMC2245901

[61] Altschul SF, Madden TL, Schäffer AA, Zhang J, Zhang Z, Miller W, et al Gapped BLAST and PSI-BLAST: a new generation of protein database search programs. Nucleic Acids Res. 1997;25: 3389–3402. 9254694 10.1093/nar/25.17.3389 PMC146917

[62] Weng J, Ma J, Fan K, Wang W. Asymmetric conformational flexibility in the ATP-binding cassette transporter HI1470/1. Biophys J. 2009;96: 1918–1930. 10.1016/j.bpj.2008.11.035 19254551 PMC2717296

[63] Kovach AR, Hoff KE, Canty JT, Orans J, Brennan RG. Recognition of U-rich RNA by Hfq from the Gram-positive pathogen Listeria monocytogenes. RNA N Y N. 2014;20: 1548–1559. 10.1261/rna.044032.113 PMC4174437 25150227

[64] Benkert P, Künzli M, Schwede T. QMEAN server for protein model quality estimation. Nucleic Acids Res. 2009;37: W510–514. 10.1093/nar/gkp322 19429685 PMC2703985

[65] Christodoulides M. Neisseria proteomics for antigen discovery and vaccine development. Expert Rev Proteomics. 2014;11: 573–591. 10.1586/14789450.2014.938640 25017717

[66] Dennehy R, McClean S. Immunoproteomics: The Key to Discovery of New Vaccine Antigens Against Bacterial Respiratory Infections. Curr Protein Pept Sci. 2012;13: 807–815. 10.2174/138920312804871184 23305366 PMC3594738

[67] Dobrindt U, Blum-Oehler G, Nagy G, Schneider G, Johann A, Gottschalk G, et al Genetic Structure and Distribution of Four Pathogenicity Islands (PAI I536 to PAI IV536) of Uropathogenic Escherichia coli Strain 536. Infect Immun. 2002;70: 6365–6372. 10.1128/IAI.70.11.6365-6372.2002 12379716 PMC130402

[68] Pawlik M-C, Hubert K, Joseph B, Claus H, Schoen C, Vogel U. The Zinc-Responsive Regulon of Neisseria meningitidis Comprises 17 Genes under Control of a Zur Element. J Bacteriol. 2012;194: 6594–6603. 10.1128/JB.01091-12 23043002 PMC3497534

[69] Sonnleitner E, Hagens S, Rosenau F, Wilhelm S, Habel A, Jäger K-E, et al Reduced virulence of a hfq mutant of Pseudomonas aeruginosa O1. Microb Pathog. 2003;35: 217–228. 14521880 10.1016/s0882-4010(03)00149-9

[70] Sittka A, Pfeiffer V, Tedin K, Vogel J. The RNA chaperone Hfq is essential for the virulence of Salmonella typhimurium. Mol Microbiol. 2007;63: 193–217. 10.1111/j.1365-2958.2006.05489.x 17163975 PMC1810395

[71] Ding Y, Davis BM, Waldor MK. Hfq is essential for Vibrio cholerae virulence and downregulates sigma expression. Mol Microbiol. 2004;53: 345–354. 10.1111/j.1365-2958.2004.04142.x 15225327

[72] Fantappiè L, Metruccio MME, Seib KL, Oriente F, Cartocci E, Ferlicca F, et al The RNA Chaperone Hfq Is Involved in Stress Response and Virulence in Neisseria meningitidis and Is a Pleiotropic Regulator of Protein Expression. Infect Immun. 2009;77: 1842–1853. 10.1128/IAI.01216-08 19223479 PMC2681778

[73] Dietrich M, Munke R, Gottschald M, Ziska E, Boettcher JP, Mollenkopf H, et al The effect of hfq on global gene expression and virulence in Neisseria gonorrhoeae. FEBS J. 2009;276: 5507–5520. 10.1111/j.1742-4658.2009.07234.x 19691497

[74] Hoang C, Ferré-D’Amaré AR. Cocrystal Structure of a tRNA Ψ55 Pseudouridine Synthase: Nucleotide Flipping by an RNA-Modifying Enzyme. Cell. 2001;107: 929–939. 10.1016/S0092-8674(01)00618-3 11779468

[75] Dorman CJ, Deighan P. Regulation of gene expression by histone-like proteins in bacteria. Curr Opin Genet Dev. 2003;13: 179–184. 12672495 10.1016/s0959-437x(03)00025-x

[76] Kwiatek A, Bacal P, Wasiluk A, Trybunko A, Adamczyk-Poplawska M. The dam replacing gene product enhances Neisseria gonorrhoeae FA1090 viability and biofilm formation. Front Microbiol. 2014;5 10.3389/fmicb.2014.00712 PMC4269198 25566225

[77] Miller HI, Kirk M, Echols H. SOS induction and autoregulation of the himA gene for site-specific recombination in Escherichia coli. Proc Natl Acad Sci U S A. 1981;78: 6754–6758. 6796964 10.1073/pnas.78.11.6754 PMC349128

[78] Piekarowicz A, Kłyż A, Majchrzak M, Adamczyk-Popławska M, Maugel TK, Stein DC. Characterization of the dsDNA prophage sequences in the genome of Neisseria gonorrhoeae and visualization of productive bacteriophage. BMC Microbiol. 2007;7: 66 10.1186/1471-2180-7-66 17615066 PMC1931599

[79] Janosi L, Shimizu I, Kaji A. Ribosome recycling factor (ribosome releasing factor) is essential for bacterial growth. Proc Natl Acad Sci. 1994;91: 4249–4253. 8183897 10.1073/pnas.91.10.4249 PMC43762

[80] Cannon JG. Conserved lipoproteins of pathogenic Neisseria species bearing the H.8 epitope: lipid-modified azurin and H.8 outer membrane protein. Clin Microbiol Rev. 1989;2: S1–S4. 2470496 10.1128/cmr.2.suppl.s1 PMC358069

[81] Bae JE, Schurig GG, Toth TE. Mice immune responses to Brucella abortus heat shock proteins: Use of baculovirus recombinant-expressing whole insect cells, purified Brucella abortus recombinant proteins, and a vaccinia virus recombinant as immunogens. Vet Microbiol. 2002;88: 189–202. 10.1016/S0378-1135(02)00101-3 12135637

[82] Kelly DF, Rappuoli R. Reverse Vaccinology and Vaccines for Serogroup B Neisseria meningitidis In: Pollard AJ, Finn A, editors. Hot Topics in Infection and Immunity in Children II. Springer US; 2005 pp. 217–223. Available: http://link.springer.com/chapter/10.1007/0-387-25342-4_15. 10.1007/0-387-25342-4_15 16107075

[83] Swanson J, Mayer LW, Tam MR. Antigenicity of Neisseria gonorrhoeae outer membrane protein(s) III detected by immunoprecipitation and Western blot transfer with a monoclonal antibody. Infect Immun. 1982;38: 668–672. 6183218 10.1128/iai.38.2.668-672.1982 PMC347790

[84] Pannekoek Y, Dankert J, van Putten JP. Construction of recombinant neisserial Hsp60 proteins and mapping of antigenic domains. Mol Microbiol. 1995;15: 277–285. 7746149 10.1111/j.1365-2958.1995.tb02242.x

[85] Knaust A, Weber MVR, Hammerschmidt S, Bergmann S, Frosch M, Kurzai O. Cytosolic Proteins Contribute to Surface Plasminogen Recruitment of Neisseria meningitidis. J Bacteriol. 2007;189: 3246–3255. 10.1128/JB.01966-06 17307854 PMC1855851

[86] Spence JM, Tyler RE, Domaoal RA, Clark VL. L12 enhances gonococcal transcytosis of polarized Hec1B cells via the lutropin receptor. Microb Pathog. 2002;32: 117–125. 10.1006/mpat.2001.0484 11855942

[87] Grifantini R, Bartolini E, Muzzi A, Draghi M, Frigimelica E, Berger J, et al Gene expression profile in Neisseria meningitidis and Neisseria lactamica upon host-cell contact: from basic research to vaccine development. Ann N Y Acad Sci. 2002;975: 202–216. 12538166 10.1111/j.1749-6632.2002.tb05953.x

[88] Williams JN, Skipp PJ, Humphries HE, Christodoulides M, O’Connor CD, Heckels JE. Proteomic Analysis of Outer Membranes and Vesicles from Wild-Type Serogroup B Neisseria meningitidis and a Lipopolysaccharide-Deficient Mutant. Infect Immun. 2007;75: 1364–1372. 10.1128/IAI.01424-06 17158897 PMC1828559

[89] Ferrari G, Garaguso I, Adu-Bobie J, Doro F, Taddei AR, Biolchi A, et al Outer membrane vesicles from group B Neisseria meningitidis Δgna33 mutant: Proteomic and immunological comparison with detergent-derived outer membrane vesicles. PROTEOMICS. 2006;6: 1856–1866. 10.1002/pmic.200500164 16456881

[90] Jacobsson K, Rosander A, Bjerketorp J, Frykberg L. Shotgun phage display—Selection for bacterial receptins or other exported proteins. Biol Proced Online. 2003;5: 123–135. 10.1251/bpo54 14569614 PMC154567

[91] Hust M, Dübel S, Schirrmann T. Selection of recombinant antibodies from antibody gene libraries. Methods Mol Biol Clifton NJ. 2007;408: 243–255. 10.1007/978-1-59745-547-3_14 18314587

[92] Stothard P. The sequence manipulation suite: JavaScript programs for analyzing and formatting protein and DNA sequences. BioTechniques. 2000;28: 1102, 1104. 10868275 10.2144/00286ir01

[93] Altschul SF, Gish W, Miller W, Myers EW, Lipman DJ. Basic local alignment search tool. J Mol Biol. 1990;215: 403–410. 10.1016/S0022-2836(05)80360-2 2231712

[94] Yu NY, Wagner JR, Laird MR, Melli G, Rey S, Lo R, et al PSORTb 3.0: improved protein subcellular localization prediction with refined localization subcategories and predictive capabilities for all prokaryotes. Bioinforma Oxf Engl. 2010;26: 1608–1615. 10.1093/bioinformatics/btq249 PMC2887053 20472543

[95] Petersen TN, Brunak S, von Heijne G, Nielsen H. SignalP 4.0: discriminating signal peptides from transmembrane regions. Nat Methods. 2011;8: 785–786. 10.1038/nmeth.1701 21959131

[96] Pettersen EF, Goddard TD, Huang CC, Couch GS, Greenblatt DM, Meng EC, et al UCSF Chimera—a visualization system for exploratory research and analysis. J Comput Chem. 2004;25: 1605–1612. 10.1002/jcc.20084 15264254

