# Supplementary figures and images for: Identification of Novel Immunogenic Proteins of Neisseria gonorrhoeae by Phage Display
Source: PLoS One. 2016 Feb 9;11(2):e0148986. doi: 10.1371/journal.pone.0148986 (PMC4747489; doi:10.1371/journal.pone.0148986)

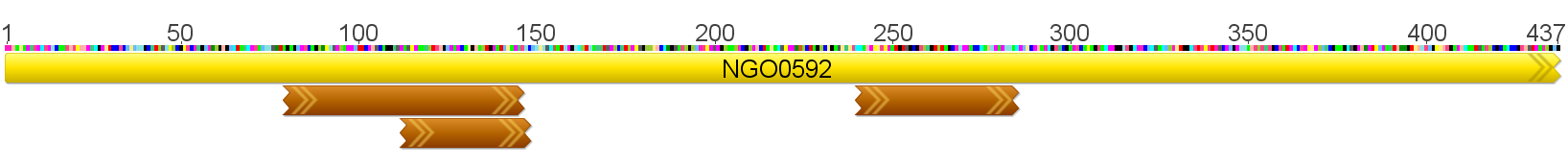

Supplement: S1 Fig — In total, three oligopeptides were identified; two of them overlapping between amino acids 79 and 148, the third in a different part of the protein spanning from amino acid residue 240 to 285. (TIF) [file pone.0148986.s001.tif]

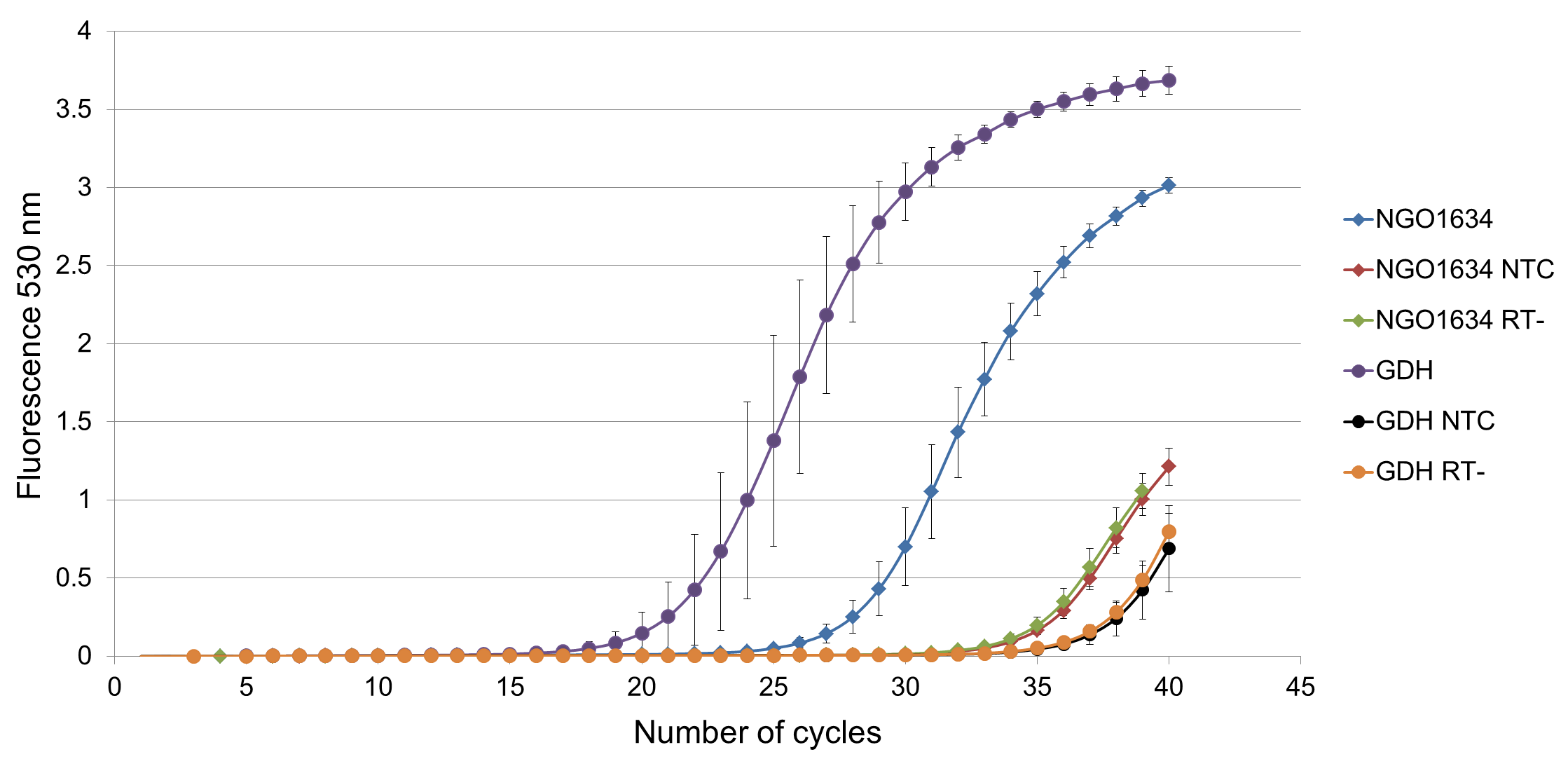

Supplement: S2 Fig — The Cp values were 27.25 ± 0.82 for NGO1634, 20.94 ± 1.7 for GDH (NGO0715) and > 35 for the controls. (TIF) [file pone.0148986.s002.tif]
